# The Influence of Age and Exercise Training Status on Left Ventricular Systolic Twist Mechanics in Healthy Males—An Exploratory Study

**DOI:** 10.3390/jcdd11100321

**Published:** 2024-10-12

**Authors:** Alexander J. Beaumont, Amy K. Campbell, Viswanath B. Unnithan, David Oxborough, Fergal Grace, Allan Knox, Nicholas F. Sculthorpe

**Affiliations:** 1 School of Science, Technology and Health, York St. John University, York YO31 7EX, UK; a.campbell@yorksj.ac.uk; 2 Institute of Clinical Exercise and Health Sciences, School of Health and Life Sciences, University of the West of Scotland, Hamilton G72, 0LH, UK; vish.unnithan@uws.ac.uk (V.B.U.); nicholas.sculthorpe@uws.ac.uk (N.F.S.); 3 Research Institute of Sport and Exercise Science, Liverpool John Moores University, Liverpool L3 3AF, UK; d.l.oxborough@ljmu.ac.uk; 4 Faculty of Health, School of Health Science and Psychology, Federation University Australia, Ballarat, VIC 3350, Australia; f.grace@federation.edu.au; 5 Exercise Science Department, California Lutheran University, Thousand Oaks, CA 91360, USA; allanknoxphd@gmail.com

**Keywords:** left ventricle, ageing, exercise, cardiac mechanics, echocardiography

## Abstract

Age-related differences in twist may be mitigated with exercise training, although this remains inconclusive. Moreover, temporal left ventricular (LV) systolic twist mechanics, including early-systolic (twist_early_), and beyond peak twist (twist_peak_) alone, have not been considered. Therefore, further insights are required to ascertain the influence of age and training status on twist mechanics across systole. Forty males were included and allocated into 1 of 4 groups based on age and training status: young recreationally active (Y_RA_, n = 9; 28 ± 5 years), old recreationally active (O_RA_, n = 10; 68 ± 6 years), young trained (Y_T_, n = 10; 27 ± 6 years), and old trained (O_T_, n = 11, 64 ± 4 years) groups. Two-dimensional speckle-tracking echocardiography was performed to determine LV twist mechanics, including twist_early_, twist_peak_, and total twist (twist_total_), by considering the nadir on the twist time-curve during early systole. Twist_total_ was calculated by subtracting twist_early_ from their peak values. LV twist_peak_ was higher in older than younger men (*p* = 0.036), while twist_peak_ was lower in the trained than recreationally-active (*p* = 0.004). Twist_peak_ is underestimated compared with twist_total_ (*p* < 0.001), and when early-systolic mechanics were considered, to calculate twist_total_, the age effect (*p* = 0.186) was dampened. LV twist was higher in older than younger age, with lower twist in exercise-trained than recreationally-active males. Twist_peak_ is underestimated when twist_early_ is not considered, with novel observations demonstrating that the age effect was dampened when considering twist_early_. These findings elucidated a smaller age effect when early phases of systole are considered, while lower LV systolic mechanics were observed in older aged trained than recreationally-active males.

## 1. Introduction

Healthy, chronological ageing is associated with a multitude of changes within the human physiological system, including the heart and major blood vessels. Even in the absence of systemic or conventional risk factors (smoking, diabetes, hypertension), the ageing heart leads to intrinsic structural and functional deteriorations [1], and together, the gross and cellular modifications within the ageing heart can impair left ventricular (LV) lusitropy and myocardial function [2].

Since pioneering descriptions of the LV rotary motion [3], the scientific community has further elucidated the unique fibre arrangements and architecture of the myocardium, which mechanistically underpins LV twisting mechanics [4]. The absolute difference between basal and apical rotation throughout systole is represented as net LV twist [4], and the twisting motion contributes towards achieving a sufficient ejection fraction [5]. Technological advancements have facilitated the detailed assessment of LV motion, including rotations and twisting during systole [4,6]. Indeed, accumulating evidence suggests that LV twist increases with advancing age [7,8,9,10]. It has been proposed that this age-related increase in twist may be the consequence of subendocardial fibrosis/dysfunction [11,12], thereby reducing the capacity of the endocardium to provide an opposing rotation to the dominant epicardium [13].

The United Kingdom government advocates modifiable lifestyle factors, such as exercise and physical activity, for preventing cardiovascular disease and preserving healthy ageing [14]. It is well established that superior exercise capacity is associated with a greater likelihood of survival [15]. The ‘masters athlete’ represents a useful model to differentiate age-related physiological declines that can be prevented from those which are inevitable [16]. Thus, the masters athlete may be viewed as having the maximum potential for preserving cardiovascular health [17] through the utilisation of a non-pharmacological, behaviour-orientated approach. A recent meta-analysis identified larger LV mass, volume, wall thicknesses, and diastolic function in masters athletes than matched controls [16], commensurate with the ‘athlete’s heart’ phenotype. Long-term exercise training appears to play an effective role in offsetting some of the detrimental changes during advancing age [17]. Although similar ejection fractions and global longitudinal strain have been reported between masters athletes and controls [16], the influence of exercise training in association with age-related systolic twist mechanics is equivocal. Contrasting findings exist as to whether older exercise-trained individuals do [10,18] or do not [19,20] display lower LV twist than their matched untrained counterparts, thus requiring more investigation.

Peak rotation and twist (apical_peak_, basal_peak_ and twist_peak_) are typically reported as the highest value achieved during systole from the ‘zero’ baseline. However, during the preceding isovolumic contraction (IVC) phase, initial electrical activation of the endocardium at the apical septal wall facilitates subendocardial shortening and subepicardial stretching [21,22,23]. This electromechanical sequence of myofibers that precedes LV ejection underpins the brief apical clockwise rotation and basal counterclockwise rotation [4,22,24,25] and is thought to be due to endocardial fibres dominating the direction of rotation [23,24]. Mechanics during this phase of the cardiac cycle may be termed the early-systolic twist (twist_early_) and early systolic apical (apical_early_) and basal (basal_early_) rotations, and may provide insight to endocardial function and by extension, age-related increase in LV twist. Although these mechanics have been scarcely considered, basal_early_ rotation has been shown to reduce with age [26,27], which may be sensitive to detect endocardial dysfunction [28]. Moreover, without equal apical_early_, basal_early_ and twist_early_ between individuals, the nadir point of LV twist on the twist-time curve will be variable. However, it is unclear if the nadir point influences the total systolic twisting during ejection since opposing apical_early_, basal_early_ and twist_early_ are ignored when determining peak values, with the possibility that the total amount (maximum degrees) of twist and rotation is therefore underestimated. This may have important ramifications when attempting to ascertain LV systolic function. As a result, calculating the total amount of counterclockwise twist (as viewed on the twist-time curve) would appropriately determine the total amount of LV twisting during ejection. Furthermore, temporal analysis of LV systolic mechanics has revealed nuanced differences in basal_early_ and apical_early_ between athlete groups [29], yet this has not been explored regarding ageing and training status.

Overall, more research is needed to determine whether chronic exercise training can mitigate age-related differences in LV twist. Additionally, it is not clear if early mechanics prior to ejection (apical_early_, basal_early_ and twist_early_) contribute to differences in observed peak values near end-systole. Therefore, exploratory data are needed to provide scope to further understand the influence of training status as a countermeasure to chronological ageing. Accordingly, this exploratory study aimed to (1) investigate the influence of healthy ageing and exercise training status on LV twist mechanics and (2) further investigate temporal systolic twist mechanics using speckle-tracking echocardiography (STE). In accordance with this, it was hypothesised that LV twist would be higher in older than younger groups yet lower in trained than untrained groups. Secondly, we hypothesised that considering twist_early_ mechanics would alter twist_peak_ and, by extension, reduce the age and training status effects.

## 2. Materials and Methods

### 2.1. Overview and Participants

Sixty-eight males were initially recruited, and standardised exclusion criteria were applied [30], leading to the exclusion of 28 participants. Twelve older (O_RA_ = 9; O_T_ = 3) adults were excluded due to the presence of (e.g., myocardial infarction, angina, stroke, and peripheral artery disease) and/or treatment (e.g., anti-hypertensives and beta-blockers) for cardiovascular diseases or type 2 diabetes mellitus. Two smokers were excluded (Y_RA_ = 2), and two participants violated pre-participation restrictions (Y_RA_ = 2). Additionally, seven trained participants were excluded due to either inconsistent training (Y_T_ = 3) or having not been training for ≥5 years (O_T_ = 4). Five participants withdrew due to personal reasons. Consequently, 40 males were included and allocated into 1 of 4 groups based on age and training status (young recreationally-active [Y_RA_], n = 9, 28 ± 5 years; young trained [Y_T_], n = 10, 27 ± 6 years; old recreationally-active [O_RA_], n = 10, 68 ± 6 years; and old trained [O_T_], n = 11, 64 ± 4 years). The overview of this study, participant baseline characteristics and inclusion/exclusion criteria have been published elsewhere [30]. Here, we present unpublished data pertaining to twist mechanics in relation to ageing and exercise training. However, see Supplementary Material File S1 for a list of baseline characteristics included for the reader’s interest. All participants provided written, informed consent before being enrolled.

As reported by Beaumont et al. [30], the Y_RA_ and O_RA_ groups were not engaged with structured exercise habits (Y_RA_, 67 ± 87 min per week, 48.5 ± 5.0 mL·kg^−1^·min^−1^; O_RA_, 63 ± 67 min per week, 34.9 ± 7.3 mL·kg^−1^·min^−1^) and all performed < 2 h per week of physical activity. In contrast, the Y_T_ (450 ± 239 min per week, 64.1 ± 7.7 mL·kg^−1^·min^−1^) group were required to have trained for at least 6 months since LV twist has been shown to increase following six months of training [31]. Y_T_ took part in running (n = 3), cycling (n = 2) and both modalities (n = 4) for 5 ± 4 years. The O_T_ (540 ± 180 minutes per week, 50.1 ± 3.6 mL·kg^−1^·min^−1^) group were included if they had trained for at least 5 years [10,32,33] and commenced training before 64 years of age, given the reported adaptations in LV compliance following exercise training in those aged 45–64 years of age but not in those aged 65 years and older [17,34]. O_T_ consisted of those involved with running (n = 5), cycling (n = 2) or both modalities (n = 4) for 34 ± 14 years, including three previous international athletes and a half marathon world champion within their age group.

### 2.2. Protocol and Experimental Procedures

#### 2.2.1. Echocardiography

Standard image acquisition techniques used in this study are presented by Beaumont et al. [30], pertaining to LV structure, conventionally derived function, and the capture of parasternal short-axis views.

#### 2.2.2. Left Ventricular Twist Mechanics

The parasternal short-axis at basal and apical levels was acquired, with the basal plane obtained as circular as possible at the level of the full mitral valve. The apex was captured without the visibility of papillary muscles [35] by tilting the transducer from an original apical 4-chamber orientation and moving slightly to the point above LV luminal obliteration [4,13]. The image with the smallest LV chamber at end-systole was selected for speckle-tracking analysis using dedicated semi-automated software (EchoPac, version 202). Aortic valve closure (AVC) was identified as end-systolic timing from the pulsed wave tracings obtained from the apical 5-chamber LV outflow tract.

**Figure 1:**
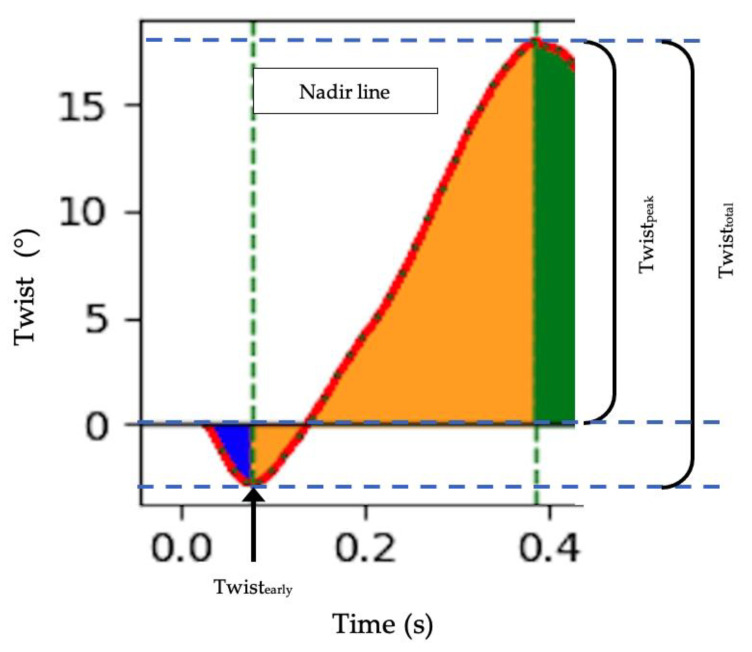
Example of identification for left ventricle twist mechanics, including twist_early_, twist_peak_ and twist_total_ on a twist-time curve during systole.

Images were recorded at a frame rate of ~71 fps for speckle-tracking analysis at both apical and basal levels. In the instance that two or more segments could not be tracked sufficiently, the image was excluded from analysis. Raw text files were imported into custom software, which applied a 1000-point cubic spline to each of the systolic and diastolic portions of the cardiac cycle (derived from AVC). The splined data were used to identify peaks in IVC during early systole and peaks in twist occurring at end-systole or early diastole. Peak clockwise basal (basal_peak_) and counterclockwise apical (apical_peak_) rotation and simultaneous net twist (twist_peak_) were identified and also scaled to LV length to determine normalised rotations and torsion, respectively [4]. Basal_early_ and apical_early_ were identified to signify counterclockwise basal and clockwise apical rotation as the highest positive and negative values during early systole prior to the subsequent rotation in the opposing direction. Likewise, twist_early_ represented the nadir point on the twist-time curve (Figure 1). To determine total rotation (apical_total_ and basal_total_) and twist (twist_total_) after taking into consideration the nadir on the rotation/twist time-curve, apical_early_, basal_early_, and twist_early_ were subtracted from their peak values, respectively (Figure 1). Time-to-peak corresponding to early and peak rotations/twist were calculated as absolute timings (milliseconds). Time displacement represented the absolute time difference between basal_peak_ and apical_peak_, and was calculated as the difference between time-to-basal_peak_ and apical_peak_ [36].

All images were acquired and analysed by a single sonographer (AB). Within-day test-retest reproducibility was conducted in 8 young individuals and was calculated using the coefficient of variation (basal_peak_, 15.1%; apical_peak_, 12.4%; twist_peak_, 14.1%). These reproducibility values align with other intra-observer data [37,38].

### 2.3. Statistical Analysis

All statistical analyses of data were conducted using jamovi (version 0.9 [39]). The influence of ageing and exercise training on markers of LV twist mechanics were analysed using two-way analysis of variance (ANOVA) to assess the main effects of age, training status, and their interaction. In the presence of a statistically significant interaction, Tukey’s post hoc test was used to explore between-group differences. Statistical significance was granted at *p* ≤ 0.050.

## 3. Results

**Figure 2:**
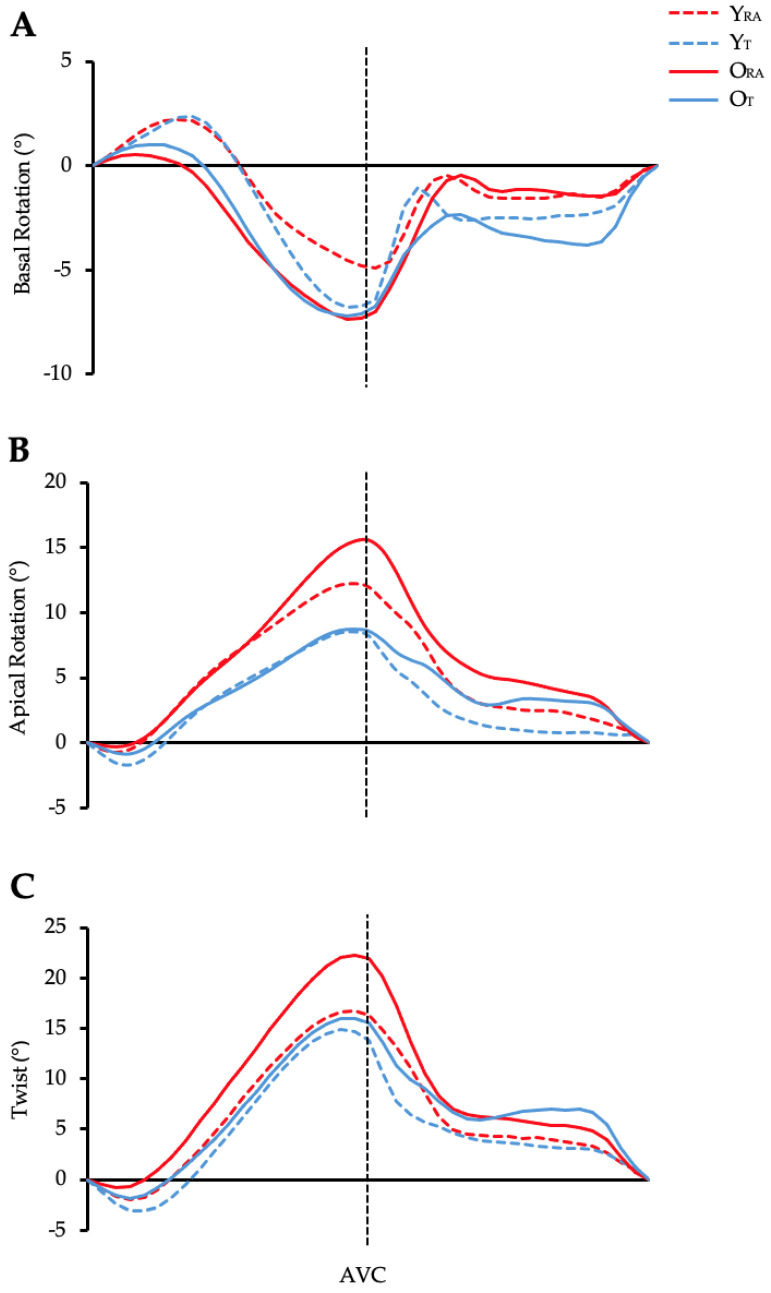
(**A**–**C**) Temporal LV rotation ((**A**)—basal rotation; (**B**)—basal rotation) and twist (**C**) across the cardiac cycle in 5% increments. AVC, aortic valve closure (end-systole [100%]). Data are presented as group mean with error bars omitted for clarity.

Data pertaining to LV structure, volumes, and function have been published previously [30]; see Supplementary Material Files S2 and S3 for the reader’s interest. Figure 2A–C illustrates average temporal LV rotations and twist between groups.

### 3.1. Left Ventricular Basal Rotation

**Figure 3:**
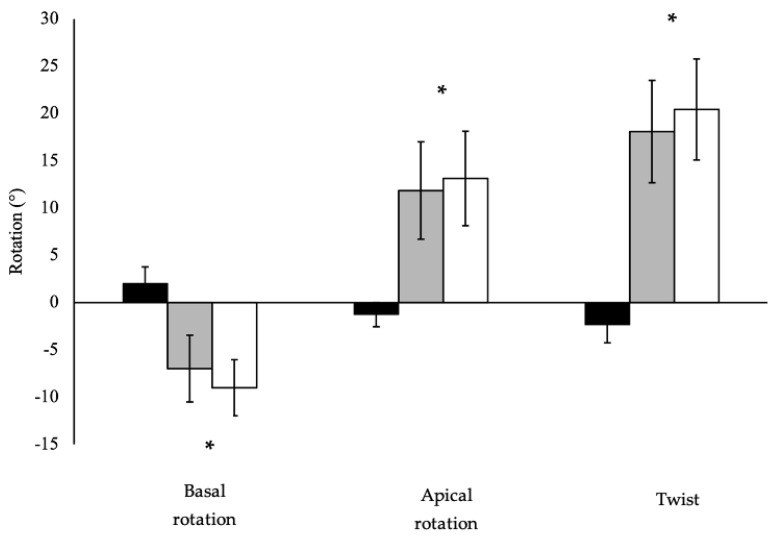
Left ventricular early (black bars), peak (grey bars) and total (white bars) rotations from apical and basal levels, and net twist for the pooled cohort (n = 40). Data are presented as mean ± standard deviation. * indicates statistically significant difference between peak and total rotations within the respective pair at *p* < 0.001. Left ventricular early rotation/twist are presented for illustrative purposes.

Pooled (n = 40) basal_peak_ was significantly lower than basal_total_ (*p* < 0.001; Figure 3). Basal rotation and respective timings are presented in Table 1 and Table 2, respectively. Older cohorts demonstrated lower basal_early_ than younger cohorts (*p* = 0.025), while basal_peak_ (*p* = 0.164), basal_total_ (*p* = 0.841) and normalised basal rotation (*p* = 0.094) demonstrated no age effect. No significant training status effects or interaction effects were observed for basal rotation for basal_early_ (*p* = 0.679; *p* = 0.742, respectively), basal_peak_ (*p* = 0.421; *p* = 0.482, respectively), basal_total_ (*p* = 0.273; *p* = 0.568, respectively) or normalised basal rotation (*p* = 0.421; *p* = 0.482, respectively). Similarly, time-to-peak basal_early_ and basal_peak_ rotation did not differ based on age (*p* = 0.720; *p* = 0.251, respectively) or training status (*p* = 0.534; *p* = 0.937, respectively), with no interaction (*p* = 0.942; *p* = 0.731, respectively).

### 3.2. Left Ventricular Apical Rotation

Pooled (n = 40) apical_peak_ was significantly lower than apical_total_ (*p* < 0.001; Figure 3). Apical rotation and respective timings are presented in Table 1 and Table 2, respectively. Apical_early_ (*p* = 0.379), apical_peak_ (*p* = 0.206), apical_total_ (*p* = 0.310), and normalised peak apical rotation (*p* = 0.105) did not differ between young and old. Similarly, apical_early_ demonstrated no significant difference in trained than recreationally-active groups (*p* = 0.069), whereas trained groups demonstrated significantly lower apical_peak_ (*p* < 0.001), apical_total_ (*p* = 0.004) and normalised peak apical rotation than recreationally-active groups (*p* < 0.001). No significant age x training interactions were observed for indices of apical_early_ (*p* = 0.393), apical_peak_ (*p* = 0.173), apical_total_ (*p* = 0.112) or normalised apical rotation (*p* = 0.201). Time-to-peak apical_early_ and apical_peak_ rotation did not differ based on age (*p* = 0.635; *p* = 0.204, respectively), or training status (*p* = 0.295; *p* = 0.545, respectively), with no interaction (*p* = 0.980; *p* = 0.563, respectively).

Time displacement did not differ based on age (*p* = 0.882) or training status (*p* = 0.648), with no interaction (*p* = 0.435, Table 2).

### 3.3. Left Ventricular Twist

**Figure 4:**
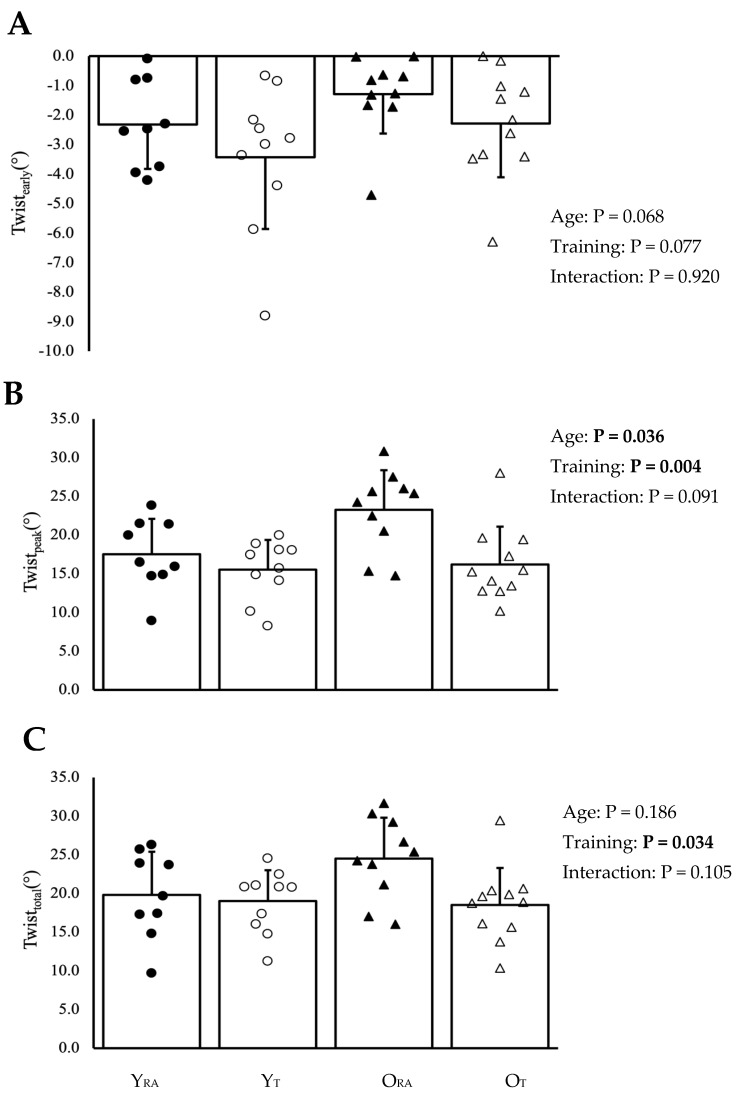
(**A**–**C**) Left ventricular twist mechanics for groups based on age and training status. Data are presented as mean ± SD, and bold *p*-values indicate statistical significance at *p* < 0.05. Y_RA_, young recreationally active; Y_T_, young trained; O_RA_, old recreationally active; O_T_, old trained.

Pooled (n = 40) twist_peak_ was significantly lower than twist_total_ (*p* < 0.001; Figure 3). Twist_early_, twist_peak_ and twist_total_ are illustrated in Figure 4A–C, respectively. Time-to-peak twist_early_ and twist_peak_ are presented in Table 2. There were no statistically significant differences in twist_early_ between older and younger groups (*p* = 0.068) or between trained and recreationally-active groups (*p* = 0.077), with no significant interaction (*p* = 0.920). Twist_peak_ was higher in older than in younger cohorts (*p* = 0.036) and lower in trained than in recreationally-active groups (*p* = 0.004), with a non-significant interaction (*p* = 0.091). In contrast, twist_total_ did not differ between young and old cohorts (*p* = 0.186) but remained significantly lower in trained than in recreationally-active groups (*p* = 0.034), with no significant interaction (*p* = 0.105). Torsion (LV twist_peak_ normalized to LV length) was significantly greater in older than younger groups (*p* = 0.009) and lower in trained than recreationally-active cohorts (*p* = 0.001), with a non-significant interaction (*p* = 0.119). Time-to-peak twist_early_ did not differ based on age (*p* = 0.865) or training status (*p* = 0.446), with no interaction (*p* = 0.890). Conversely, twist_peak_ was later in older than younger groups (*p* = 0.018), and there was no significant difference in time to twist_peak_ (*p* = 0.710) based on training status or an interaction (*p* = 0.402).

## 4. Discussion

The principal findings from this exploratory study were that LV twist_peak_ was higher in older than younger males, yet twist_peak_ was lower in trained than recreationally-active males. Moreover, the findings of this study suggest that twist_early_ may influence twist_peak_ during systole, such that there was less difference in LV twisting between young and older aged groups, as reflected by twist_total_. This is, to our knowledge, the first documentation that early mechanics prior to ejection may contribute to observed peak values near end-systole.

### 4.1. Age-Related Differences in LV Twist

Twist_peak_ and torsion were higher in older than younger groups, which aligns with the known ageing process [7,8,9,10,11]. Moreover, our present observations of lower basal_early_ with ageing, yet similar apical_early_, agree with others [26,27]. Additionally, we noted a smaller twist_early_ in older than younger males, which approached statistical significance.

An imbalance between epicardial and endocardial fibres from subendocardial fibrosis/dysfunction is a recurrent proposal for the age-associated increase in twist_peak_ [11,12]. This theory is plausible since less opposing rotation within the endocardium would permit greater dominance of epicardial rotation, leading to heightened overall twist [40]. Examination of mechanics prior to twist_peak_ has provided more insight into these age-related changes, potentially related to the endocardium. During IVC, endocardial shortening and epicardial stretching produce basal_early_ and apical_early_ [22]. Thus, since endocardial mechanical activity is responsible for basal_early_ [24,27], it is possible that ageing may reduce endocardial shortening during IVC, explaining less basal_early_ that we and others have reported in older than in younger groups [27]. This result is further extended by our findings of lower twist_early_, while not achieving statistical significance, in older groups than in younger. Altered basal_early_ may, therefore, represent a sensitive marker to endocardial dysfunction in this context associated with advancing age [28]. Still, it is unclear why apical_early_ was not different between ages, which is also due to endocardial shortening [27].

It has also been proposed that temporal alignment of apical and basal rotation may contribute to an age-related increase in simultaneous twist [27]. Results from this study are not in agreement with those from van Dalen et al. [27] since relative timings of apical and basal rotations did not differ with age, nor did the absolute time displacement between respective peak timings. Possible reasons for the discrepancies are not clear, although the acquisition of displacement time may provide some insight. An inherent limitation of 2D speckle tracking means that apical and basal images are acquired separately in different cardiac cycles, and thus, absolute heart rates and loading conditions could influence the timing alignment between rotations captured at the base and apex.

### 4.2. LV Twist and Exercise Training Status

Twist_peak_, twist_total,_ apical_peak_ and apical_total_ were lower in exercise trained groups in this study compared to recreationally-active participants. These observations, in consideration of the differences with healthy ageing, suggest exercise training status in older age mitigates an age-related difference in twist. Existing data concerning ageing, exercise, and twist are in their infancy, but findings to date are conflicting. Our observations disagree with others who have observed similar resting twist between middle-aged trained and untrained men [19,20], yet concur with reports of lower twist in middle-aged athletes than age-matched controls [10,18]. Although all studies have included participants from endurance-based and highly dynamic modalities [41], such as cycling, triathlon, speed skating, running and swimming, the influence of specific activities will warrant further consideration. Indeed, the static components of highly dynamic, endurance activities vary [41], which has shown to influence the training status related differences in LV twist mechanics, albeit in younger cohorts [42]. In the current study, the twist and torsion observations were likely due to lower apical_peak_ in O_T_, since basal_peak_ was unaltered; whereas Maufrais et al. [10] attributed the lower twist to basal adaptations only. The reason for this regional disparity is unclear, however, reduced twist has been more commonly accompanied by lower apical rotation and not the base, at least in younger athletes [42,43]. The causes for altered LV mechanics with chronic exercise across all ages have not been fully elucidated. It would seem unlikely that preserved longitudinal subendocardial functioning in O_T_ is responsible since layer-specific shortening was similar to O_RA_ [30]. In addition to LV strain, elite cyclists showed lower apical rotation and twist in both endocardial and epicardial layers [44].

It is not clear at this stage whether the lower twist and apical rotation reflect a reduction in systolic function or represent a functional reserve for exercise capacity [43]. Without a cohort design, it is difficult to determine if the lower twist is a response to chronic and extended exercise training. Indeed, 39 months of rowing training produced a similar twist and apical rotation as a baseline, which was preceded by an initial increase due to acute exercise [45]. Therefore, in our study, the long-term training of older adults may have lowered twist further. From a geometry perspective, less LV twist was related to larger mean wall thickness [43], and a reduction in twist following a more extended period of exercise training may have been associated with cellular hypertrophy [45]. Greater indexed mean wall thickness and LVmass in trained than recreationally-active [30] would support this proposition, with the latter potentially negating the requirement for a bigger systolic contribution to LV ejection in accordance with a larger stroke volume and lower heart rate [30]. Still, lower apical rotation in those with superior aerobic capacity occurred without LV structural adaptation [46] and thus, hypertrophy may not be a prerequisite for altered twist mechanics. Furthermore, changes in fibre angle are known to influence twist [47], as is the sphericity index [48]. However, the sphericity index did not differ between groups in this study [30]; therefore, fibre rearrangement angulation may require further exploration in the absence of differences in the sphericity index. Altered autonomic function following exercise training characterised by heightened parasympathetic and reduced sympathetic activity may influence LV mechanics [49], corresponding to higher and lower LV twist following dobutamine and esmolol infusion, respectively [49]. Although the mechanisms of adaptation require clarification, these data do indicate that chronic exercise training in older adults produces LV adaptations that present a younger phenotype than their untrained counterparts.

### 4.3. Temporal LV Systolic Twist

We extend existing studies by documenting for the first time that twist_early_ may be an important consideration when interpreting twist_peak_. We demonstrated that twist_peak_ is significantly underestimated compared with twist_total_, which considers twist_early_. Therefore, when assessing the age-related differences in LV twist mechanics, twist_total_, produces a different statistical outcome between young and old adults than twist_peak_. Thus, twist_total_ may reflect a narrowing in the amount of LV twisting between young and old. Although, caution must be applied when interpreting our results as we do not suggest that the age effect on twist is abolished because of twist_early_, nor do we propose that when assessing twist_total_, pump function is equal between young and old males. Instead, it is to our knowledge the first documentation of a reduction in the ageing-related difference in twist through important considerations of mechanics during early systole. These data would suggest that conventional approaches to quantifying twist_peak_ and the anticipated differences between young and old may be inconsistent and, in some cases, underestimated since the preceding counterclockwise rotation during early-systole, below ‘zero’ has not been accounted for. Therefore, individual variations in twist_early_ may, in turn, impact the magnitude of difference in twist_peak,_ in this case, between different age groups. If older and younger ages are characterised by smaller and larger twist_early_, respectively, the onset of counterclockwise twist may begin at different points in relation to the ‘zero line’. The subsequent peak_twist_ taken from the ‘zero line’ may, therefore, be over- or under-estimated. In turn, this may have implications for the appropriate quantification of LV function, although the contribution of twisting occurring before ‘zero’ to LV ejection requires clarification. Simultaneous assessment of LV twisting and structure/volume, like others have reported recently for strain-volume loops [50], may provide further insight into the contribution that twist_total_ may have on systolic function. Although systole and diastole are linked by LV twist mechanics [13], twist_total_ could be less informative in the context of early diastolic function since the maximum amount of twist would have already occurred. The impact of exercise training status appeared to be less influenced by twist_total_, which remained significantly lower in trained than recreationally-active groups. Together, these data highlight the importance of considering the temporal sequence of LV twist, including all phases of systole to reflect the nadir point on the twist-time curve.

### 4.4. Limitations

Study limitations pertaining to the cohorts used in this study have been reported previously [30]. Although the study sample size is small, we employed stringent inclusion criteria to facilitate separate homogenous groups related to healthy ageing and exercise training. Post-hoc power analysis between O_RA_ and O_T_ for twist_peak_ identified a calculated effect size of 1.4 (Cohen’s D) and achieved a power of 87%, suggesting sufficient statistical power in the current study. However, given the small sample size used in this study, results should still be interrupted with caution and replication work is needed to further elucidate the age- and exercise-related differences in twist mechanics using a larger sample size. Our healthy cohort may explain why twist was obtained in all participants (100%) since reports that only 19% (n = 206) of older males (69 ± 6 years) with various medical histories had adequate LV short-axis images at both the base and apex to enable LV twist [51]. Only men were included in the present study based on sex-dependent LV mechanics with ageing [9,52,53], and as a consequence, findings should not be generalised to the female population. There exists a paucity of data on the female community and it should, therefore, be investigated in relation to ageing and exercise. Without an experimental study design, we cannot ascertain causality for training and age-related changes in LV twist mechanics, nor group-based differences which are attributed to factors beyond assessment in this study (e.g., genetics). However, since a longitudinal study in O_T_ athletes across multiple decades (31 ± 11 years of training) is not feasible, we used a cross-sectional study with training-based inclusion criteria for the O_T_ to reflect a cohort of chronically trained males that is heterogenous to their recreationally active counterparts (O_RA_). We did not normalise LV twist simultaneously with LV length/structure, which future work may wish to do so when determining the contribution of LV twist to ejection across phases of the cardiac cycle, similar to strain-volume loops reported recently [50].

## 5. Conclusions

Findings from this exploratory study showed that while LV twist is higher in older than younger aged groups, LV twist is lower in exercise-trained than in recreationally-active males. Novel observations demonstrated that when considering twist_early_ to reflect the nadir point on the twist-time curve, the difference in the amount of LV twisting between young and old males is reduced.

## Figures and Tables

**Table 1:** Left ventricular twist mechanics among age and exercise training groups.

	Young		Old		*p*-Value		
	Recreationally Active (Y_RA_)	Trained (Y_T_)	Recreationally Active (O_RA_)	Trained (O_T_)	Age	Training	Interaction
Basal_early_ rotation (°)	2.6 ± 2.0	2.7 ± 2.1	1.2 ± 1.1	1.6 ± 1.6	**0.025**	0.679	0.742
Basal_peak_ rotation (°)	−5.4 ± 2.7	−7.0 ± 2.5	−7.6 ± 3.0	−7.7 ± 4.3	0.164	0.421	0.482
Basal_total_ rotation (°)	−8.1 ± 2.8	−9.7 ± 1.9	−8.8 ± 3.5	−9.3 ± 3.5	0.841	0.273	0.568
Normalised basal rotation (°/cm)	−0.6 ± 0.3	−0.7 ± 0.3	−0.9 ± 0.4	−0.9 ± 0.5	0.094	0.564	0.560
Apical_early_ rotation (°)	−0.9 ± 0.7	−2.0 ± 1.4	−0.9 ± 1.5	−1.3 ± 1.2	0.379	0.069	0.393
Apical_peak_ rotation (°)	12.6 ± 3.9	9.5 ± 3.0	16.3 ± 6.1	9.3 ± 4.0	0.206	**<0.001**	0.173
Apical_total_ rotation (°)	13.5 ± 3.9	11.5 ± 2.9	17.2 ± 6.2	10.6 ± 4.0	0.310	**0.004**	0.112
Normalised apical rotation (°/cm)	1.4 ± 0.4	1.0 ± 0.3	1.9 ± 0.7	1.0 ± 0.5	0.105	**<0.001**	0.201
Torsion (°/cm)	1.9 ± 0.5	1.6 ± 0.4	2.6 ± 0.6	1.8 ± 0.5	**0.009**	**0.001**	0.119

Data are presented as mean ± SD. Bold values indicate statistical significance at *p* < 0.05.

**Table 2:** Left ventricular mechanical timings during early and peak systole between age and exercise training groups.

	Young		Old		*p*-Value		
	Recreationally Active (Y_RA_)	Trained (Y_T_)	Recreationally Active (O_RA_)	Trained (O_T_)	Age	Training	Interaction
Time to basal_early_ rotation (ms)	272 ± 77	295 ± 103	263 ± 99	281 ± 125	0.720	0.534	0.942
Time to basal_peak_ rotation (ms)	348 ± 50	343 ± 37	362 ± 73	369 ± 48	0.251	0.937	0.731
Time to apical_early_ rotation (ms)	215 ± 76	247 ± 107	230 ± 83	260 ± 93	0.635	0.295	0.980
Time to apical_peak_ rotation (ms)	363 ± 36	385 ± 71	397 ± 48	397 ± 60	0.204	0.545	0.563
Time displacement (ms)	−16 ± 52	−42 ± 60	−35 ± 66	−28 ± 77	0.882	0.648	0.435
Time to twist_early_ (ms)	233 ± 72	260 ± 112	242 ± 86	261 ± 96	0.865	0.446	0.890
Time to twist_peak_ (ms)	350 ± 41	355 ± 34	389 ± 42	374 ± 31	**0.018**	0.710	0.402

Data are presented as mean ± SD. Bold values indicate statistical significance at *p* < 0.05.

## Data Availability

Data are not openly available due to restrictions upon ethical approval. Please contact the corresponding author for data requests.

## Supplementary Materials

The following supporting information can be downloaded at: https://www.mdpi.com/article/10.3390/jcdd11100321/s1, Supplementary Material Table S1: Baseline physical and exercise characteristics including training habits and maximal oxygen uptake in young recreationally active (Y_RA_), young trained (Y_T_), old recreationally active (O_RA_) and old trained (O_T_) participants; Table S2: Left ventricular structure, geometry and volumes in young recreationally active (Y_RA_), young trained (Y_T_), old recreationally active (O_RA_) and old trained (O_T_) participants; Table S3: Ultrasonic measures of conventional left ventricular (LV) systolic and diastolic function in young recreationally active (Y_RA_), young trained (Y_T_), old recreationally active (O_RA_) and old trained (O_T_) participants.
