# Supplementary material for: The Influence of Age and Exercise Training Status on Left Ventricular Systolic Twist Mechanics in Healthy Males—An Exploratory Study
Source: J Cardiovasc Dev Dis. 2024 Oct 12;11(10):321. doi: 10.3390/jcdd11100321 (PMC11508667; doi:10.3390/jcdd11100321)
Supplement: Supplementary file 1 [file jcdd-11-00321-s001.zip › SUPPLEMENTARY MATERIAL FILE S1.pdf]

SUPPLEMENTARY MATERIAL 1

Table 1 Baseline physical and exercise characteristics including training habits and maximal oxygen uptake in young recreationally active (Y_{RA}), young trained (Y_T), old recreationally active (O_{RA}) and old trained (O_T) participants.

	Young		Old		*P* value		
	Recreationally active (Y_{RA})	Trained (Y_T)	Recreationally active (O_{RA})	Trained (O_T)	Age	Training	Interaction
Number of participants	9	10	10	11			
Age (years)	28 ± 5 (21 – 35)	27 ± 6 (18 – 36)	68 ± 6 (60 – 80)	64 ± 4 (59 – 70)	**<0.001**	0.21	0.46
Exercise characteristics							
Minutes per week	67 ± 87	450 ± 239	63 ± 67	540 ± 180	0.41	**<0.001**	0.37
Training years	-	5 ± 4	-	34 ± 14			
Training start age (years)	-	23 ± 8	-	31 ± 11			
Height (cm)	177 ± 4	180 ± 8	175 ± 8	173 ± 4	**0.02**	0.65	0.31
Mass (kg)	80.5 ± 7.8	75.0 ± 9.3	82.0 ± 20.2	67.0 ± 5.6	0.40	**0.01**	0.22
Systolic blood pressure (mmHg)	131 ± 9	126 ± 10	131 ± 11	132 ± 16	0.47	0.63	0.34
Diastolic blood pressure (mmHg)	77 ± 8	68 ± 7	74 ± 8	74 ± 8	0.47	0.09	0.06
Mean arterial pressure (mmHg)	95 ± 8	87 ± 7	93 ± 8	94 ± 10	0.38	0.18	0.11
Resting heart rate (beats·min^{-1})	61 ± 11	49 ± 11	54 ± 5	47 ± 7	0.14	**0.001**	0.35

Rate pressure product (mmHg/beats·min^{-1})	8049 ± 1810	6084 ± 1063	7038 ± 571	6231 ± 1073	0.26	**<0.001**	0.13
Body surface area (m^2)	1.99 ± 0.11	1.94 ± 0.15	1.98 ± 0.27	1.79 ± 0.09	0.17	**0.03**	0.22
Body mass index (kg·m^2)	25.5 ± 1.7	23.1 ± 2.2	26.8 ± 5.1	22.3 ± 1.6	0.83	**<0.001**	0.31
$\dot{V}O_{2max}$ (L·min^{-1})	3.9 ± 0.4	4.8 ± 0.7	2.7 ± 0.4	3.4 ± 0.3	**<0.001**	**<0.001**	0.40
$\dot{V}O_{2max}$ (mL·kg^{-1}·min^{-1})	48.5 ± 5.0	64.1 ± 7.7	34.9 ± 7.3	50.1 ± 3.6	**<0.001**	**<0.001**	0.93
Age predicted $\dot{V}O_{2max}$ (%)	92 ± 9	121 ± 12	94 ± 21	128 ± 8	0.32	**<0.001**	0.55

$\dot{V}O_{2max}$, maximal oxygen uptake. $p \leq 0.050$. * vs Y_{RA}; † vs. Y_T; ‡ vs. O_{RA}. Data presented as means ± SD or range in parentheses. Data previously published in Beaumont et al., (2020).

References

Beaumont A, Campbell A, Unnithan V, et al (2020) Long-term athletic training does not alter age-associated reductions of left-ventricular mid diastolic lengthening or expansion at rest. Eur J Appl Physiol. https://doi.org/10.1007/s00421-020-04418-1
