# Supplementary material for: The Influence of Age and Exercise Training Status on Left Ventricular Systolic Twist Mechanics in Healthy Males—An Exploratory Study
Source: J Cardiovasc Dev Dis. 2024 Oct 12;11(10):321. doi: 10.3390/jcdd11100321 (PMC11508667; doi:10.3390/jcdd11100321)
Supplement: Supplementary file 1 [file jcdd-11-00321-s001.zip › SUPPLEMENTARY MATERIAL FILE S2.pdf]

SUPPLEMENTARY MATERIAL 2

Table 2 Left ventricular structure, geometry and volumes in young recreationally active (Y_{RA}), young trained (Y_T), old recreationally active (O_{RA}) and old trained (O_T) participants.

	Young		Old		*P* value		
	Recreationally active (Y_{RA})	Trained (Y_T)	Recreationally active (O_{RA})	Trained (O_T)	Age	Training	Interaction
IVS (mm)	9.7 ± 1.0	9.7 ± 1.0	9.6 ± 1.0	10.5 ± 1.1	0.29	0.18	0.21
IVS index (mm/(m^2)$^{0.5}$)	6.9 ± 0.7	7.0 ± 0.5	6.8 ± 0.7	7.8 ± 0.8	0.07	**0.02**	0.06
LVEDD (mm)	48.9 ± 2.0	51.6 ± 3.0	46.4 ± 2.7	49.6 ± 3.9	**0.03**	**0.004**	0.79
LVEDD index (mm/(m^2)$^{0.5}$)	34.7 ± 1.3	37.1 ± 1.5	31.6 ± 4.6	37.0 ± 2.1	0.08	**<0.01**	0.10
PWT (mm)	10.1 ± 0.8	10.5 ± 1.4	9.7 ± 1.3	10.2 ± 1.0	0.32	0.24	0.90
PWT index (mm/(m^2)$^{0.5}$)	7.2 ± 0.5	7.5 ± 0.8	6.9 ± 1.0	7.6 ± 0.8	0.74	**0.04**	0.53
MWT (mm)	9.9 ± 0.6	10.1 ± 1.1	9.7 ± 0.9	10.3 ± 0.9	0.97	0.14	0.44
MWT (mm/(m^2)$^{0.5}$)	7.0 ± 0.4	7.3 ± 0.6	6.9 ± 0.7	7.7 ± 0.7	0.40	**0.01**	0.14
LVM (g)	177 ± 21	201 ± 44	157 ± 26	192 ± 37	0.20	**0.01**	0.60
LVM (g/m^2)	89 ± 9	103 ± 16	80 ± 13	107 ± 17	0.59	**<0.001**	0.16
LVM (g/(m^2)$^{2.7}$)	37 ± 4	41 ± 6	35 ± 6	44 ± 8	0.87	**0.01**	0.19
RWT	0.41 ± 0.02	0.39 ± 0.03	0.42 ± 0.05	0.42 ± 0.05	0.11	0.52	0.51
LV length (mm)	91 ± 5	96 ± 7	89 ± 5	91 ± 6	**0.05**	0.07	0.33
LV length (mm/(m^2)$^{0.5}$)	64 ± 3	69 ± 6	63 ± 3	68 ± 4	0.31	**0.002**	0.86

Concentricity (g/mL)$^{0.667}$)	8.3 ± 1.2	7.5 ± 1.1	8.1 ± 1.5	7.9 ± 0.8	0.85	0.20	0.33
Sphericity index	1.9 ± 0.1	1.9 ± 0.2	1.9 ± 0.1	1.8 ± 0.2	0.81	0.35	0.31
LVOT diameter (mm)	21 ± 1	23 ± 2	21 ± 1	22 ± 2	0.25	0.09	0.42
LVEDV (mL)	98 ± 9	139 ± 25	85 ± 15	123 ± 20	**0.02**	**<0.001**	0.82
LVEDV (mL/(m^2)$^{1.5}$)	35 ± 4	52 ± 9	32 ± 5	51 ± 6	0.30	**<0.001**	0.58
LVESV (mL)	39 ± 2	58 ± 13	33 ± 9	47 ± 14	**0.02**	**<0.001**	0.60
LVESV (mL/(m^2)$^{1.5}$)	14 ± 2	21 ± 4	12 ± 2	19 ± 5	0.09	**<0.001**	0.99
LVOT CSA (cm^2)	3.5 ± 0.5	4.0 ± 0.7	3.4 ± 0.4	3.6 ± 0.7	0.25	0.07	0.42
VTI (cm)	21.6 ± 4.6	25.1 ± 3.5	19.5 ± 2.6	24.8 ± 5.0	0.34	**0.002**	0.49
SV (mL)	75 ± 14	102 ± 24	67 ± 13	89 ± 21	0.10	**<0.001**	0.69
SV index (mL/(m^2)$^{1.5}$)	27 ± 5	37 ± 7	24 ± 3	37 ± 9	0.47	**<0.001**	0.59
$\dot{Q}$ (L.min^{-1})	4.57 ± 1.25	4.77 ± 0.63	3.64 ± 0.85	4.12 ± 0.79	**0.009**	0.24	0.63
$\dot{Q}$ index (L.min/(m^2)$^{1.5}$)	1.65 ± 0.50	1.79 ± 0.30	1.30 ± 0.20	1.71 ± 0.30	0.06	**0.01**	0.22

CSA, cross-sectional area; IVS, interventricular septum; LVEDD, left ventricular end-diastolic diameter; PWT, posterior wall thickness; MWT, mean wall thickness; LVM, left ventricular mass; RWT, relative wall thickness; LV, left ventricular; LVOT, left ventricular outflow tract; VTI, velocity time integral; LVEDV, left ventricular end-diastolic volume; LVESV, left ventricular end-systolic volume; SV, stroke volume; $\dot{Q}$, cardiac output. Data are means ± SD. $P \leq 0.050$. * vs Y_{RA}; † vs. Y_T; ‡ vs. O_{RA}. Data previously published in Beaumont et al., (2020).

References

Beaumont A, Campbell A, Unnithan V, et al (2020) Long-term athletic training does not alter age-associated reductions of left-ventricular mid diastolic lengthening or expansion at rest. Eur J Appl Physiol. https://doi.org/10.1007/s00421-020-04418-1
