# Supplementary material for: The Influence of Age and Exercise Training Status on Left Ventricular Systolic Twist Mechanics in Healthy Males—An Exploratory Study
Source: J Cardiovasc Dev Dis. 2024 Oct 12;11(10):321. doi: 10.3390/jcdd11100321 (PMC11508667; doi:10.3390/jcdd11100321)
Supplement: Supplementary file 1 [file jcdd-11-00321-s001.zip › SUPPLEMENTARY MATERIAL FILE S3.pdf]

SUPPLEMENTARY MATERIAL 3

Table 3 Ultrasonic measures of conventional left ventricular (LV) systolic and diastolic function in young recreationally active (Y_{RA}), young trained (Y_T), old recreationally active (O_{RA}) and old trained (O_T) participants.

	Young		Old		*P* value		
	Recreationally active (Y_{RA})	Trained (Y_T)	Recreationally active (O_{RA})	Trained (O_T)	Age	Training	Interaction
Systolic function							
FS (%)	33 ± 3	30 ± 6	35 ± 3	33 ± 6	0.16	0.11	0.52
EF (%)	60 ± 4	59 ± 3	61 ± 6	61 ± 6	0.22	0.55	0.81
Septal s’ (cm/s)	9 ± 1	8 ± 1	8 ± 1	8 ± 1	**0.04**	0.71	0.94
Lateral s’(cm/s)	10 ± 3	12 ± 3	9 ± 2	9 ± 3	**0.03**	0.36	0.57
Average s’ (cm/s)	10 ± 2	10 ± 2	9 ± 1	9 ± 2	**0.03**	0.52	0.52
Average s’ index ((cm/s)/cm)	1.04 ± 0.17	1.05 ± 0.21	0.95 ± 0.15	0.95 ± 0.21	0.11	0.94	0.94
Diastolic function							
E-wave (cm/s)	73 ± 15	73 ± 11	61 ± 13	59 ± 14	**0.004**	0.72	0.83
A-wave (cm/s)	40 ± 10	38 ± 7	56 ± 7	54 ± 12	**<0.001**	0.62	0.96
E/A	1.98 ± 0.74	2.01 ± 0.56	1.13 ± 0.29	1.15 ± 0.42	**<0.001**	0.89	0.98
Septal e’(cm/s)	10 ± 3	12 ± 3	7 ± 1	8 ± 2	**<0.001**	0.09	0.51

Lateral e’ (cm/s)	13 ± 4	17 ± 2 *	9 ± 2 * †	10 ± 2 †	**<0.001**	**0.001**	**0.04**
Septal a’ (cm/s)	8 ± 2	6 ± 1	9 ± 1	9 ± 1	**<0.001**	0.09	0.09
Lateral a’ (cm/s)	7 ± 2	7 ± 2	9 ± 1	9 ± 1	**<0.001**	0.42	0.42
Average e’ (cm/s)	12 ± 3	15 ± 2	8 ± 2	9 ± 2	**<0.001**	**0.01**	0.10
Average a’ (cm/s)	8 ± 2	7 ± 2	10 ± 1	10 ± 1	**<0.001**	0.12	0.12
Average e’ index ((cm/s)/cm)	1.27 ± 0.34	1.53 ± 0.15	0.89 ± 0.17	0.97 ± 0.20	**<0.001**	**0.02**	0.20
Average a’ index ((cm/s)/cm)	0.85 ± 0.18	0.68 ± 0.22	1.05 ± 0.14	1.03 ± 0.10	**<0.001**	0.09	0.13
Average e’/a’	1.58 ± 0.68	2.45 ± 0.64 *	0.87 ± 0.23 * †	0.95 ± 0.27 * †	**<0.001**	**0.01**	**0.02**
Average E/e’	6.66 ± 1.51	4.99 ± 0.78	7.81 ± 0.98	6.94 ± 1.31	**0.03**	0.10	0.13

FS, fractional shortening; EF, ejection fraction; s’, systolic tissue velocity; E-wave, early diastolic mitral inflow velocity; A-wave, late diastolic mitral inflow velocity; E/A, ratio of early-to-late mitral inflow velocity; e’, early diastolic tissue velocity; a’, late diastolic tissue velocity; e’/a’, ratio of early-to-late diastolic tissue velocity; E/e’, ratio of early mitral inflow velocity-to-early diastolic tissue velocity. Data are means ± SD. $P \leq 0.050$. * vs Y_{RA}; † vs. Y_T; ‡ vs. O_{RA}. Data previously published in Beaumont et al., (2020).

References

Beaumont A, Campbell A, Unnithan V, et al (2020) Long-term athletic training does not alter age-associated reductions of left-ventricular mid diastolic lengthening or expansion at rest. Eur J Appl Physiol. https://doi.org/10.1007/s00421-020-04418-1
